# Gene-expression profiles in lung adenocarcinomas related to chronic wood smoke or tobacco exposure

**DOI:** 10.1186/s12931-016-0346-3

**Published:** 2016-04-20

**Authors:** Alette Ortega-Gómez, Claudia Rangel-Escareño, Camilo Molina-Romero, Eleazar Omar Macedo-Pérez, Alejandro Avilés-Salas, Alejandra Lara-García, Gerardo Alanis-Funes, Rubén Rodríguez-Bautista, Alfredo Hidalgo-Miranda, Oscar Arrieta

**Affiliations:** Thoracic Oncology Unit, Instituto Nacional de Cancerología (INCan), Mexico City, Mexico; Translational Medicine Laboratory, INCan, Mexico City, Mexico; Computational Genomics Department, Instituto Nacional de Medicina Genómica (INMEGEN), Mexico City, Mexico; Department of Pathology, INCan, Mexico City, Mexico; Department of Radiotherapy, INCan, Mexico City, Mexico; Cancer Genomics Laboratory, INMEGEN, Mexico City, Mexico; Postgraduate Unit, Faculty of Medicine, Universidad Nacional Autónoma de México (UNAM), Mexico City, Mexico

**Keywords:** Non-small cell lung carcinoma, Wood smoke exposure, Gene expression profiles, Tobacco smoke, Microarray analysis

## Abstract

**Background:**

Tobacco-smoke is the major etiological factor related to lung cancer. However, other important factor is chronic wood smoke exposure (WSE). Approximately 30 % of lung cancer patients in Mexico have a history of WSE, and present different clinical, pathological and molecular characteristics compared to tobacco related lung cancer, including differences in mutational profiles. There are several molecular alterations identified in WSE associated lung cancer, however most studies have focused on the analysis of changes in several pathogenesis related proteins.

**Methods:**

Our group evaluated gene expression profiles of primary lung adenocarcinoma, from patients with history of WSE or tobacco exposure. Differential expression between these two groups were studied through gene expression microarrays.

**Results:**

Results of the gene expression profiling revealed 57 statistically significant genes (*p* < 0.01). The associated biological functional pathways included: lipid metabolism, biochemistry of small molecules, molecular transport, cell morphology, function and maintenance. A highlight of our analysis is that three of the main functional networks represent 37 differentially expressed genes out of the 57 found. These hubs are related with ubiquitin C, *GABA(A)* receptor-associated like protein; and the *PI3K/AKT* and *MEK/ERK* signaling pathways.

**Conclusion:**

Our results reflect the intrinsic biology that sustains the development of adenocarcinoma related to WSE and show that there is a different gene expression profile of WSE associated lung adenocarcinoma compared to tobacco exposure, suggesting that they arise through different carcinogenic mechanisms, which may explain the clinical and mutation profile divergences between both lung adenocarcinomas.

## Background

Lung cancer is the first cause of death from cancer [1]. Approximately 85 % of diagnosed patients present Non-Small Cell Lung Cancer (NSCLC), and adenocarcinoma is the most frequent histological type. Despite efforts, innovations, and progress in diagnosis and treatment, 5-year overall survival is approximately 15 % with high mortality rates [2]. Tobacco smoking is the main risk of lung cancer. Other factors include pulmonary tuberculosis, genetic susceptibility, exposure to secondhand smoke, asbestos and radon [3]. In Mexico, the crude mortality rate of lung cancer is 6.68 per 10^5^ individuals, representing nearly 9000 cases per year, most of them presenting metastatic stage at diagnosis [4, 5].

Nowadays about 15 % of lung cancer in men and 53 % in women is not associated to smoking [6]. Besides, due to the impact of tobacco control policies, a bigger percentage of non-smoking patients with lung cancer is expected in the following years. According to cancer statistics from the USA, lung cancer death rates declined 36 %, from 1990 to 2011, among males and 11 %, between 2002 and 2011, among females due to reduced tobacco use as a result of increased awareness of the health hazards of smoking and the implementation of comprehensive tobacco control [2]. There have been reports of a doubling in the annual incidence of lung cancer in never smokers, identifying as well that non-smoker NSCLC patients tend to be female and young [7, 8]. Regarding mortality, never smokers present lung cancer death rates greater in men than in women and a large fraction of cases have no identified risk factors [9]. Meanwhile former smokers present an increased risk of lung cancer but cumulative risk decreases with earlier smoking cessation compared to smokers who continue smoking [10].

Chronic wood smoke exposure (WSE) is related to obstructive pulmonary disease in developing, European and American countries [11, 12]. Wood dust has also been identified as a human carcinogen and a risk factor for lung cancer [3, 13]. Wood byproducts such as benzene, 1-butadiene, formaldehyde and acetaldehyde, are well-known carcinogens [14]. For more than 50 years, WSE has been associated with an increased risk of lung cancer as compared with pulmonary tuberculosis, interstitial lung disease and various pulmonary conditions (OR: 1.9; 95 % confidence interval (CI): 1.1–3.5) after adjusting for age, education, socioeconomic status and tobacco smoke exposure [13]. In Mexico, approximately 16 % of the population has long-term exposure to wood smoke for residential heating and/or cooking, and 30 % of lung cancers are associated with WSE [5, 15]. Molecular assays have shown up-regulation and phosphorylation of p53 in WSE related lung cancer [16]. Moreover, WSE is associated with macrophage dysfunction and an increase in the activity of metalloproteinases, like *MMP-2* and *MMP-9*, which could be related to lung injury in chronic obstructive pulmonary disease and have a role in the physiopathology of lung cancer [17].

Ethnical origins and different risk factors for lung cancer might explain the distinct mutation profiles, as in the case of epidermal growth factor receptor (*EGFR)* and *KRAS* for Asians, Caucasians and Latins [18–20]. Our group previously reported a high rate of treatment response and a better outcome in patients with WSE related lung cancer treated with *EGFR*-Tyrosine Kinase Inhibitors (TKIs) [21]. We have further described that WSE related lung cancer is associated with an older age at diagnosis, adenocarcinoma histology, pleural effusion, high prevalence of *EGFR* mutations (55.4 %) and a low prevalence of *KRAS* mutation (6 %), compared to patients with smoking history [15]. These situations indicate clear differences in the molecular and clinical evolution of WSE related lung cancer compared with tobacco associated lung cancer.

In order to further analyze the molecular differences observed in WES-related lung cancer, the objective of our work was to compare the genetic expression profile of lung adenocarcinoma in patients with WSE or a smoking history.

## Methods

### Experimental design

This study used clinical, longitudinal, prospective, observational and analytical cohorts with the selection of a non–probabilistic sample type. The protocol was approved by the Scientific and Bioethical committees of the Instituto Nacional de Cancerología (INCan, 008102510M1, CB451).

### Patients and tissue samples

**Fig. 1:**
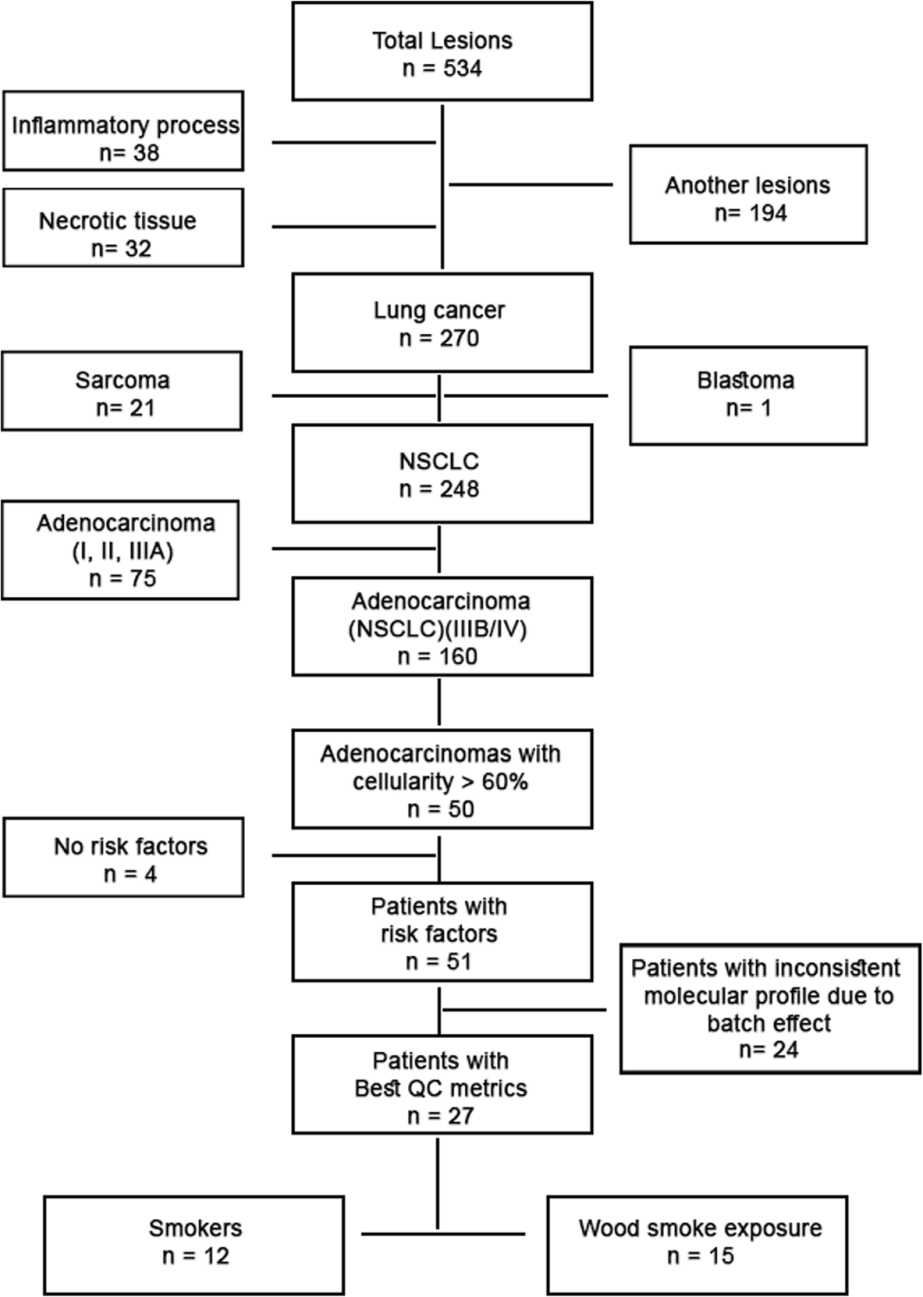
Consort

Patients admitted to the INCan with a pulmonary lesion suggestive of primary lung cancer were prospectively biopsied from January 2008 to June 2011. After informed consent, tissue was obtained by computer tomography-guided tru-cut (Care fusion, San Diego, CA, USA) from the clinically suspected primary tumor. Data were excluded from the analysis if there was no histological diagnosis, a different type of primary cancer was present, or if the pathology report indicated a histology different from lung adenocarcinoma. The patients with histologically confirmed advanced lung adenocarcinoma (stages III B and IV) were eligible for inclusion in the study (Fig. 1).

A complete medical history that included a detailed history of smoking, wood smoke exposure and a physical examination was obtained. Tumor specimens were collected at the time of diagnosis. WSE was defined as exposure to fumes resulting from burning wood in fireplaces and wood stoves for ≥ 4 h per day for ≥ 5 years. The WSE exposure index was calculated as the average number of hours spent on cooking daily per the total number of years spent cooking [22]. A smoker was defined as being someone having a lifetime exposure of more than 100 cigarettes [6]; the tobacco-smoking index was calculated by multiplying the number of cigarette packs consumed per day by the number of years spent smoking [15].

### RNA isolation and RNA preparation for microarrays

Primary tumor core-biopsy was performed prior to any treatment and snap-frozen in nitrogen for RNA extraction. A trained pathologist confirmed histological diagnosis and quantified tumor cell percentage.

The procedure for extraction and purification of total RNA from tissue (up to 5 mg tissue) was done using RNeasy Micro Kit (QIAGEN, Germany) (cat. 217084). RNA integrity was evaluated by capillary electrophoresis using the Agilent Bioanalyzer 2100 (Agilent Technologies, Santa Clara, CA). Samples with RNA integrity number (RIN) of six or higher were included for microarray analysis.

### RNA amplification and expression microarray analysis

Gene expression analysis was done using the Affymetrix GeneChip® Human Gene 1.0 ST Array System, which evaluates the expression of 28,869 different genes. Sample processing was done following the manufacturer’s instructions.

### Strategy for microarray gene-expression analysis

Statistical analysis for differential expression was conducted using R and Bioconductor. Background correction for non-specific hybridization was performed with Robust Multiarray Average (RMA) [23] which uses a fairly complex statistical model that supposes both additive and multiplicative noise components. After background correction to the individual probes, quantile normalization [24] was applied, both steps are implemented in the oligo package [25]. Normalized and corrected probes are summarized into probe sets using the median polish algorithm, which is a type of robust 2-way ANOVA, where one factor is the array and the other is the probe set. The algorithm is robust to outliers, making single probes with large values are down-weighted. Batch correction was also applied to all samples using combat in the sva package [26]. Differential expression was identified through linear models implemented in the limma package [27], genes were selected as significant according to two summary statistics: *p*-value (<10^−2^) and fold- changes larger than 1.2 in absolute values.

For the biological networks and functional analysis, we used QIAGEN’s Ingenuity Pathway Analysis (IPA®, QIAGEN Redwood City, http://www.ingenuity.com/). IPA was used to identify gene-signaling pathways that were involved in biological processes of WSE versus tobacco exposure. Networks of these genes were algorithmically generated based on their connectivity and assigned a score. The score ranks networks according to how relevant they are to the input genes not necessarily to the quality or significance of the network. The network indicates the molecular relationships between genes/gene products. Node color indicates up- or down-regulation and intensity is associated to degree of regulation. It is important to remark that uncolored genes were not identified as differentially expressed in our experiment, however, IPA integrate those into the computationally generated networks based on the collected evidence indicating a relevance to this network.

### Identification of differentially expressed genes

Significant changes in gene expression were selected according to *p*-values <10^−2^ and fold changes in absolute values larger than 1.2. Differences in gene expression are shown in volcano plots representing fold changes in log_2_ base along the x-axis and the level of trust in the form of –log_10_(*p*-values) along the y-axis. A value of 2 on the y-axis represents our cut-off of 10^−2^ (top right and left corners). Heat maps show gene profiles clustered, result of an unsupervised hierarchical clustering of genes significantly different (*p* < 0.01) between patients with different risk factors.

## Results

Overall 53 tumor samples were collected, and 29 samples were suitable for gene expression analysis. Two samples with suitable material were excluded due to patients’ history of asbestos exposure and thoracic radiotherapy. The 27 remaining samples included 12 patients with an exclusive history of tobacco exposure and 15 patients with exclusive WSE history (Fig. 1).

### Clinical and molecular results

The clinical characteristics of the 27 patients included in the microarray analysis are: mean age 62.9 ± 11.7 years, 55.6 % (15/27) were females, WSE was present on 55.6 % of patients (15/27) and 44.4 % (12/27) had tobacco exposure (Table 1). *EGFR* mutational status was statistically significant (*p* = 0.003); 53 % (8/15) of patients presented positive *EGFR* mutation status in the WSE group (Table 2).

**Table 1:** Baseline clinical pathological and molecular characteristics (*N* = 27)

Variable	% (n/N)
Gender	
Female	55.6 (15/27)
Male	44.4 (12/27)
Age	
Mean (±SD)	62.96 ± 11.7*
Wood smoke exposure	
Present	55.6 (15/27)
Absent	44.4 (12/27)
WES index	
Median (IQR)	104 ± 10
Tobacco-smoking index	
Median (IQR)	14.1 ± 14.2
Smoking exposure	
Present	44.4 (12/27)
Absent	55.6 (17/27)
Stage	3.7 (1/27)
IIIB	
IV	81.5 (22/27)
Recurrent	14.8 (4/27)
ECOG	
0–1	74.1 (20/27)
2–4	25.9 (7/27)
*EGFR* mutation	
Positive	33.3 (9/27)

*Abbreviations:* *NSCLC* non–small-cell lung cancer, *SD* standard deviation, *ECOG PS* eastern cooperative oncology group performance status, *EGFR* epidermal growth factor receptor, *WSE* wood smoke exposure

*Kolmogorov-Smirnov test *p* value: 0.20

**Table 2:** Clinical and molecular characteristics related to exposure history in all patients

Variable	WSE (*N* = 15)	Smoking exposure (*N* = 12)	P*
Gender			
Female	12	5	0.49
Male	3	7	
Age Mean (±SD)	63 (11.88)	62.9 (12.12)	0.89
^a^EGFR mutation (*N* = 9)			
Positive	8	1	
Negative	7	11	0.003
Stage			
IIIB	0	1	
IV	13	9	
Recurrent	2	2	0.70

*Non-parametric test: Mann-Whitney U test

^a^9/27 patients had EGFR mutation

### On differentially expressed genes in WSE compared with tobacco smoke exposure

**Fig. 2:**
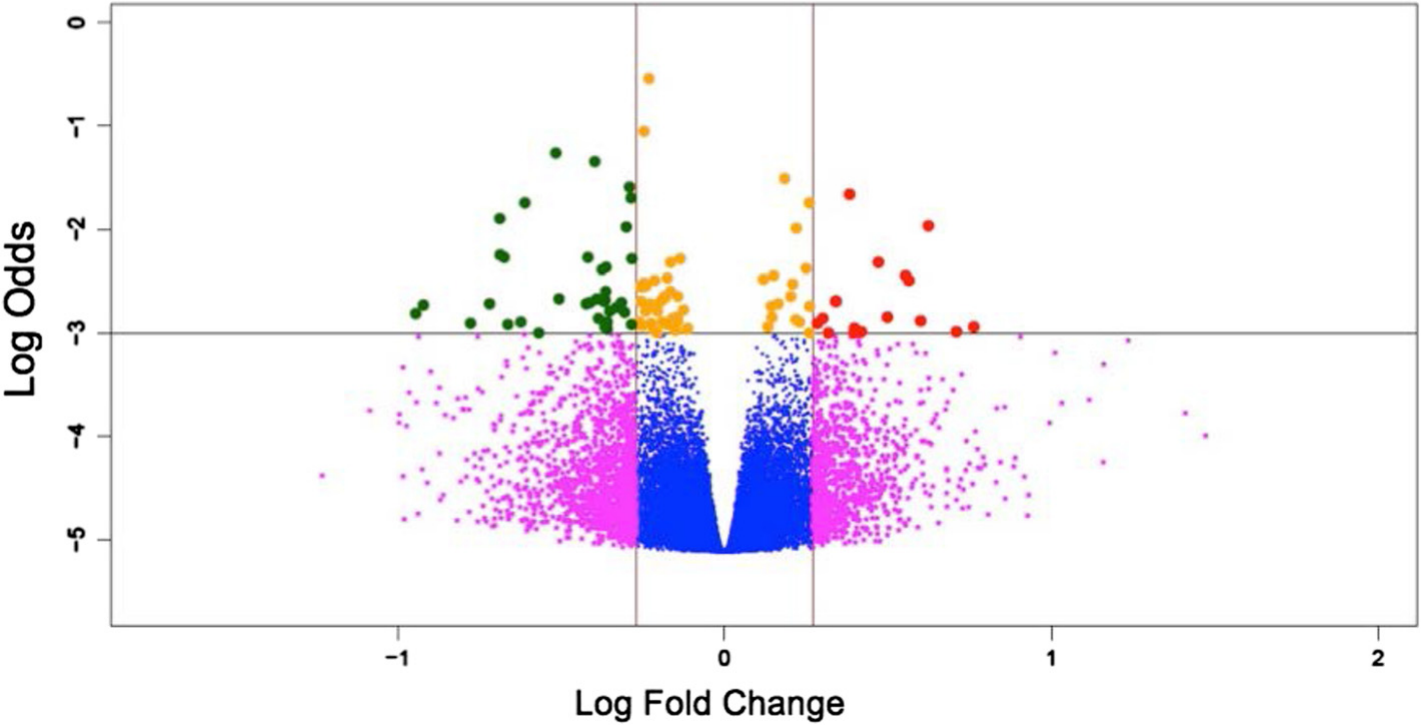
Volcano Plot showing gene differential expression of patients with adenocarcinoma and history of wood smoke exposure vs. tobacco smoke exposure. Fold changes are represented in log2 base along the x-axis and the level of trust in the form of –log_10_ (*p* < 0.001) along the y-axis. The cut off value of 10^−2^ used is 2 on the y-axis (top right and left corners). Red and green dots represent up and down-regulated genes respectively: Fold change (≥1.2) and significance level (*p* < 0.001). Other colors: yellow indicates significant but low fold change, magenta shows not statistically robust changes and blue shows low fold change with low statistical significance

**Fig. 3:**
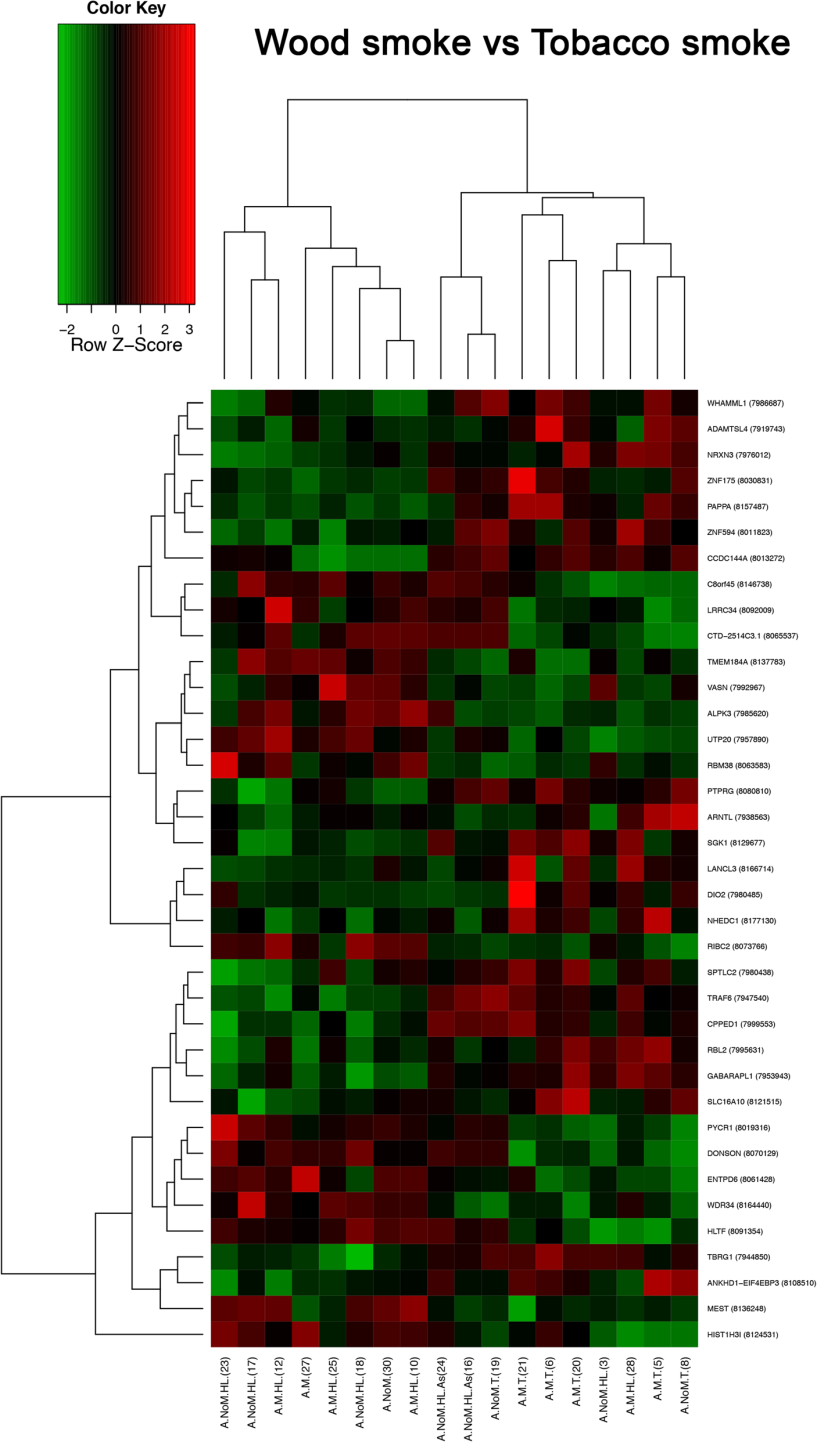
Heat map result of an unsupervised hierarchical clustering of genes significantly different (*p* < 0.01) of NSCLC adenocarcinoma exposed to wood smoke versus tobacco smoke. Each column represents a patient and each row a gene. The heat map indicates the level of gene expression. Red: high expression; Green: Low expression

Figures 2 and 3 show differences in gene expression, the gene profile shown in the clustered heat map displays significantly different genes (*p* < 0.01) between patients with WSE versus patients with a tobacco smoking history.

The comparison of adenocarcinomas from patients exposed to tobacco smoke versus WSE revealed that both groups can be separated based on the differential expression of 57 genes (*p* < 0.01), 35 up-regulated and 22 down-regulated (Fig. 3).

Enrichment and functional analysis through biological networks was conducted using IPA software. The top functional networks were related to five different biological categories as follows: *Lipid metabolism*; *Biochemistry of small molecules*; *Transport of molecules*; *Cell morphology*; *Function and cell maintenance*. Table 3 lists the up- and down-regulated genes with significant changes in expression from patients with NSCLC exposed to tobacco smoke versus WSE (*p* < 0.01). The two main categories with greater differences were *transport of molecules and cell function* and maintenance.

**Table 3:** List of genes with changed expression that are significantly (*p* < 0.01) over- and under-regulated in patients with NSCLC exposed to wood smoke versus tobacco (five IPA biological functions categories)

Symbol	Log ratio	*p*-value	Lipid metabolism	Small mol. bioch.	Mol. transport	Cell morphology	Cell. func. and main.
Upregulated genes							
HIST1H3I	0.763	9.86E-03	✓	✓	✓		
DONSON	0.554	3.82E-03	✓	✓	✓		
ENTPD6	0.472	2.98E-03	✓	✓	✓		
RIBC2	0.383	8.19E-04	✓	✓	✓		
VASN	0.286	9.15E-03	✓	✓	✓		
ASL	0.251	3.38E-03	✓	✓	✓		
DMC1	0.165	6.49E-03	✓	✓	✓		
ATP4B	0.12	4.14E-03	✓	✓	✓		
MEST	0.601	8.79E-03				✓	✓
TMEM184A	0.34	6.13E-03				✓	✓
ATG9A	0.262	6.82E-03				✓	✓
PYCR1	0.499	8.28E-03			✓		✓
RBM38	0.301	8.38E-03			✓		✓
HNRNPD	0.219	8.57E-03			✓		✓
ZNF239	2.60E-01	9.77E-04			✓		✓
Downregulated genes							
SPTLC2	−0.412	6.28E-03	✓	✓	✓		
TBRG1	−0.374	3.42E-03	✓	✓	✓		
ZNF175	−0.358	8.97E-03	✓	✓	✓		
ZNF594	−0.351	7.40E-03	✓	✓	✓		
TEX12	−0.125	7.24E-03	✓	✓	✓		
SLC16A10	−0.777	9.14E-03				✓	✓
GABARAPL1	−0.61	9.62E-04				✓	✓
PAPPA	−0.516	3.63E-04				✓	✓
MED31	−0.261	8.96E-03				✓	✓
NDUFAF1	−0.254	6.22E-03				✓	✓
CCDC144NL	−0.224	9.26E-03				✓	✓
CNTN4	−0.189	9.02E-03				✓	✓
NXPE2	−0.145	9.07E-03				✓	✓
RCOR3	−0.143	8.15E-03				✓	✓
SGK1	−0.718	6.48E-03			✓		✓
PTPRG	−0.686	2.63E-03			✓		✓
DIO2	−0.623	8.90E-03			✓		✓
RBL2	−0.369	6.19E-03			✓		✓
ARNTL	−0.366	9.77E-03			✓		✓
TRAF6	−0.329	6.87E-03			✓		✓
MBP	−0.246	4.43E-03			✓		✓

**Fig. 4:**
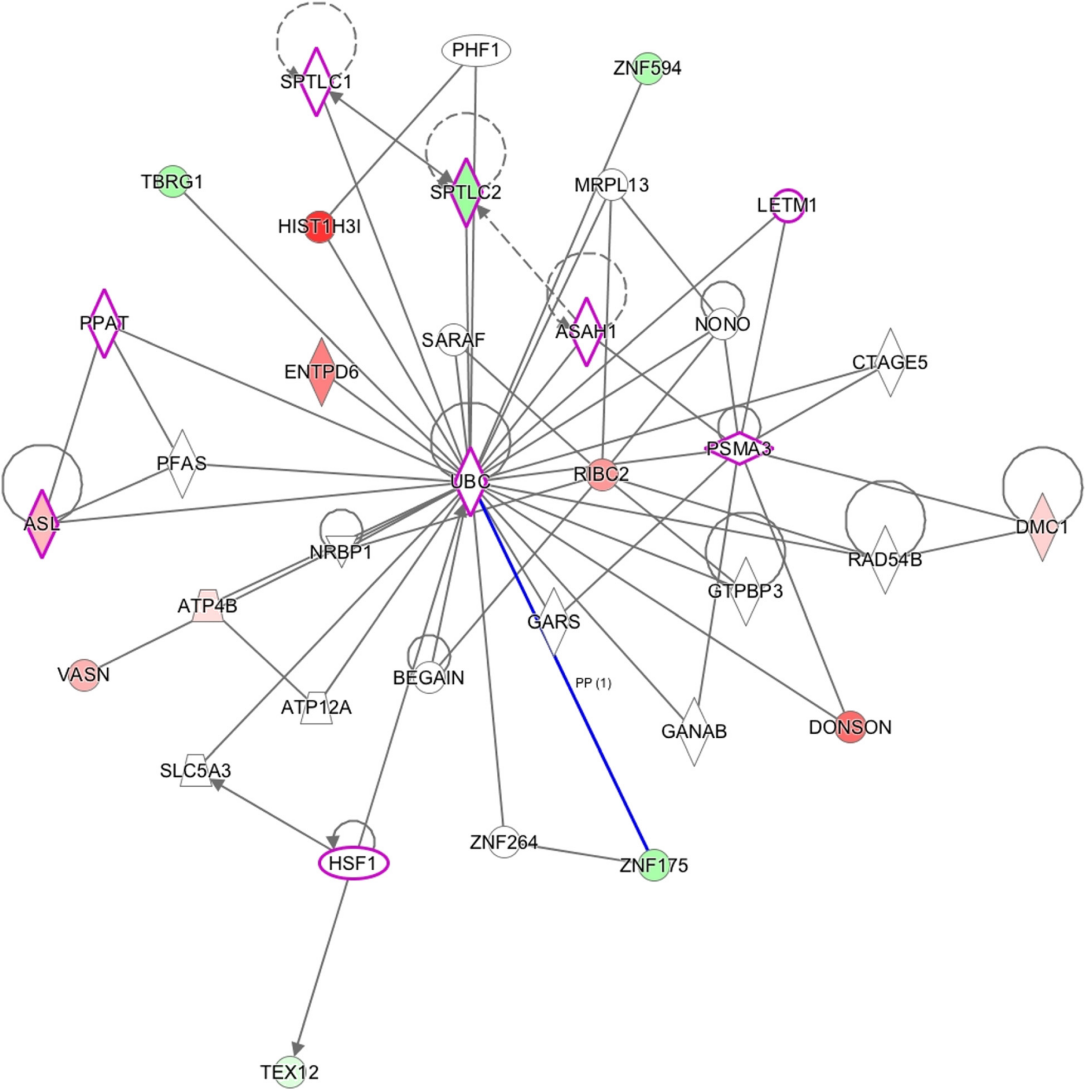
Network 1 (Score: 28/Ratio: 0.353/*p* value: 1.48E-24). Genes (*p* < 0.01) in wood smoke exposure in NSCLC around the UBC gene. Red mark: up-regulated genes. Green mark: down-regulated genes. The node shapes denote enzymes (), phosphatases (), kinases (), peptidases (), G-protein coupled receptor (), transmembrane receptor (), cytokines (), growth factor (), ion channel (), transporter (), translation factor (), nuclear receptor (), transcription factor () and other ()

**Fig. 5:**
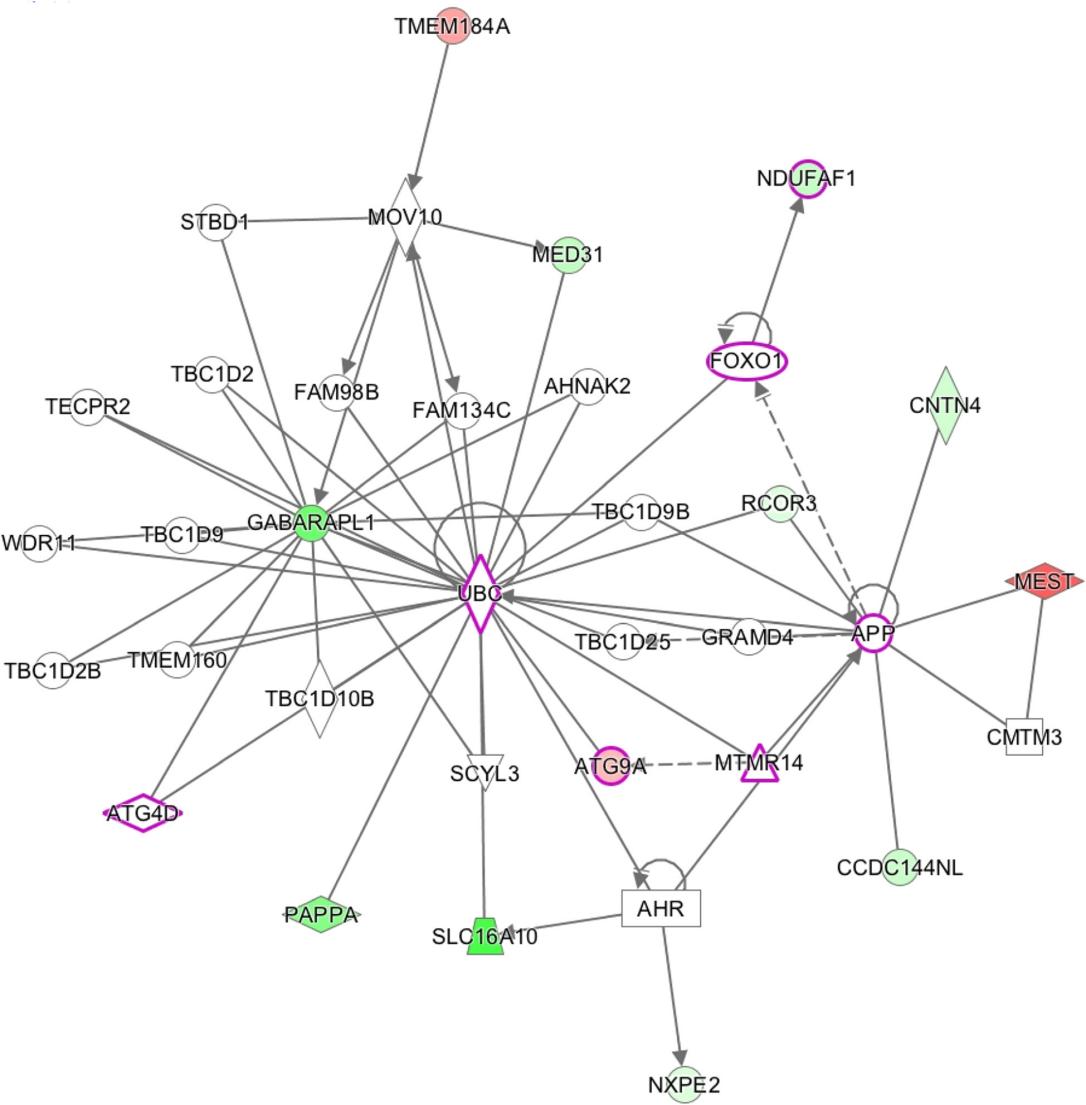
Network 2 (Score: 28/Ratio: 0.375/*p* value: 6.11E-25). Genes (*p* < 0.01) in wood smoke exposure in NSCLC around the GABARAPL1 gene. Red mark: up-regulated genes. Green mark: down-regulated genes. The node shapes denote enzymes (), phosphatases (), kinases (), peptidases (), G-protein coupled receptor (), transmembrane receptor (), cytokines (), growth factor (), ion channel (), transporter (), translation factor (), nuclear receptor (), transcription factor () and other ()

**Fig. 6:**
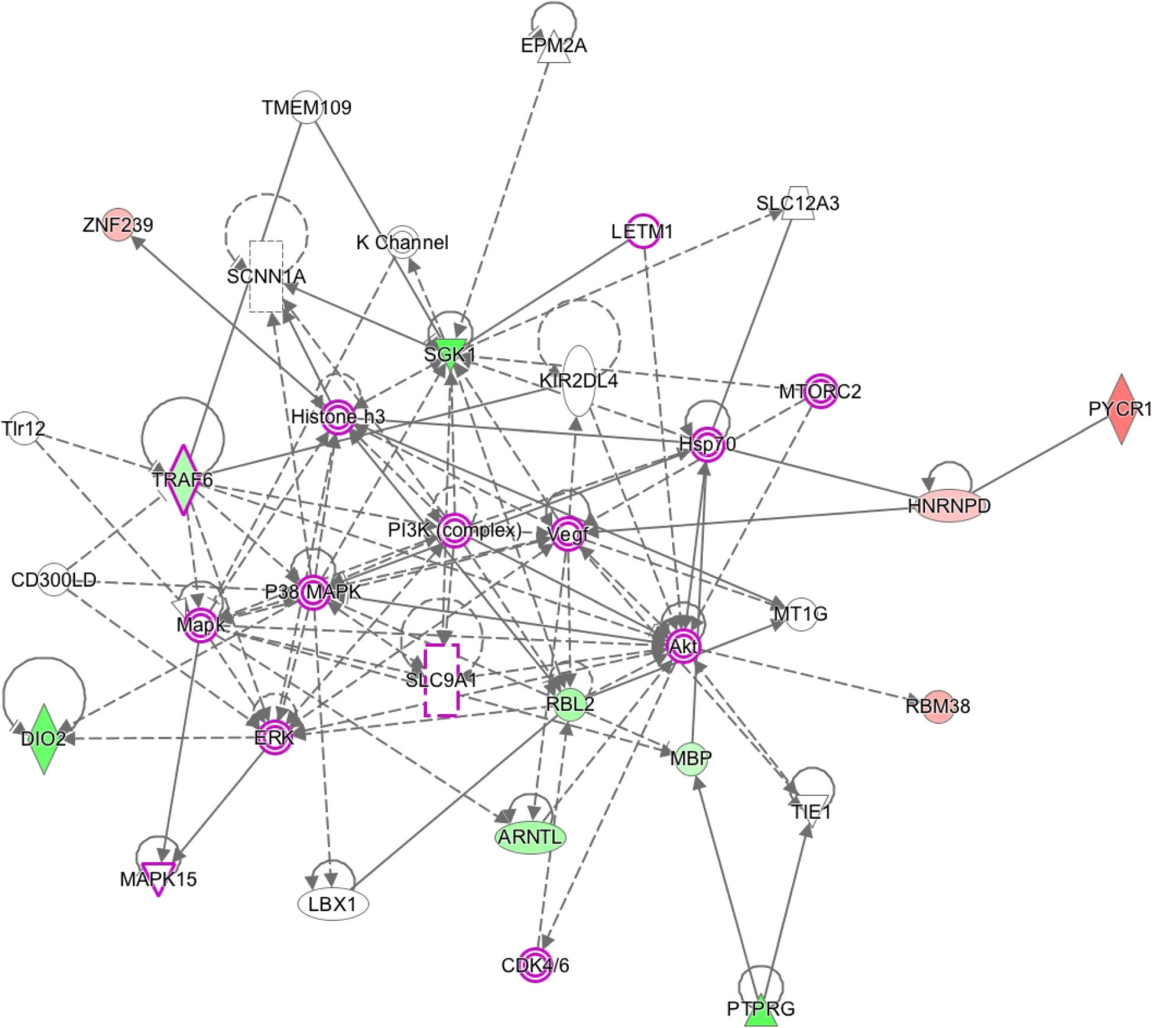
Network 3 (Score: 26/Ratio: 0.134/*p* value: 2.09E-17). Genes (*p* < 0.01) in wood smoke exposure in NSCLC around the PI3K/AKT and MEK/ERK signaling pathways. Red mark: up-regulated genes. Green mark: down-regulated genes. The node shapes denote enzymes (), phosphatases (), kinases (), peptidases (), G-protein coupled receptor (), transmembrane receptor (), cytokines (), growth factor (), ion channel (), transporter (), translation factor (), nuclear receptor (), transcription factor () and other ()

**Fig. 7:**
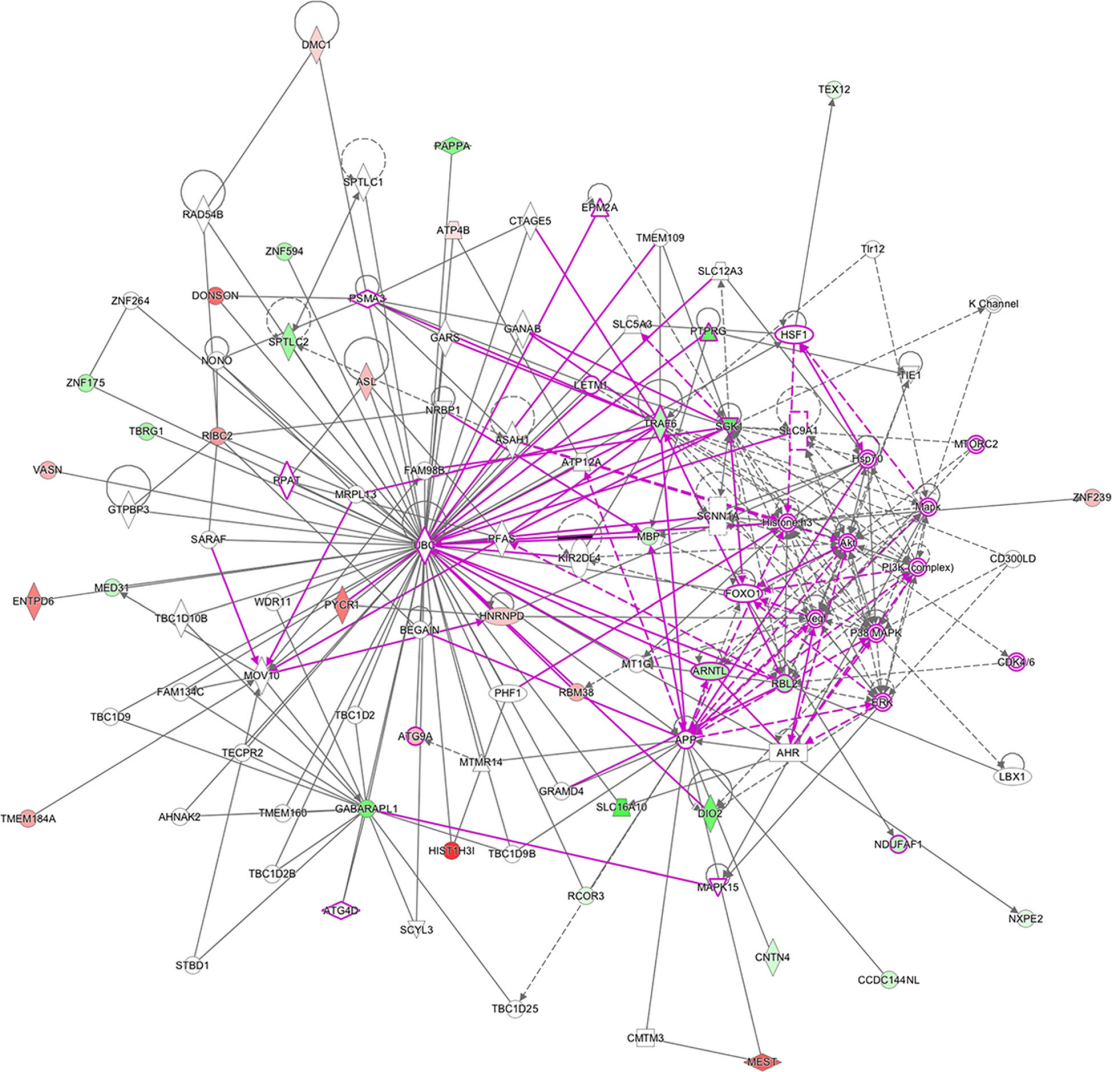
Overlapping networks and related genes (*p* < 0.01) in wood smoke exposure in NSCLC. Red mark: up-regulated genes. Green mark: down-regulated genes. Purple lines reflect the sites of intersections between our study genes (*p* < 0.01) and the main canonical networks (PI3K/AKT and MEK/ERK) associated with NSCLC. The node shapes denote enzymes (♦), phosphatases (), kinases (), peptidases (), G-protein coupled receptor ( ), transmembrane receptor ( ), cytokines (), growth factor (), ion channel (), transporter ( ), translation factor (), nuclear receptor (), transcription factor () and other ()

Three functional networks are shown, these networks involve the majority of the differentially expressed genes (37/57) and have: Ubiquitin C (UBC, score 28, Fig. 4), GABA(A) receptor-associated like protein (GABARAPL1, score 28, Fig. 5) and PI3K/AKT and MEK/ERK genes (score 26, Fig. 6) as main hubs of the network. Moreover, when the networks were overlapped, all up and down regulated genes appear around the PI3K/AKT and MEK/ERK signaling pathways (Fig. 7).

## Discussion

Although the majority of lung cancer occurs in smokers, 25 % of worldwide lung cancer occurs in life long never smokers [28], being the 7^th^ largest cause of cancer-related mortality in this group [29], presenting a wide-ranging geographic incidence and risk factors such as asbestos, air pollution, radon, arsenic compounds, cadmium, chromium, ionizing radiation and WSE [30]. Additionally, molecular profiles observed in lung cancer are critically different among smokers and non-smokers particularly identified in genes such EGFR, KRAS, P53 and ALK [31]. In the case of WSE, there have been association with NSCLC and adenocarcinoma histology, EGFR mutations, a reverse association with KRAS mutations and higher response to EGFR-TKIs [15] making it a distinctive disease entity inside the group of never smokers which would be a good candidate for personalized diagnostic and therapeutic approaches. Therefore, lung cancer associated to WSE presents unique characteristics that make it a distinctive entity of disease within the group of never smokers; thus, it could be a good candidate for personalized diagnostic and therapeutic approaches.

There is evidence of differential expression profiles associated with the bronchial epithelium of tobacco-smokers that sustains carcinogenesis [32], as well as the determination of tobacco-smoke transcriptional changes in oncogenes and anti-oncogenes [33]. Our study shows that the gene expression profiling of samples from patients with WSE is different from patients using tobacco.

Our group has previously reported that lung cancer related to tobacco smoke and WSE exhibits different clinical and pathological characteristics that may be related to different mechanisms, and this is reflected in their response rate and overall survival in NSCLC patients [15]. However, in the present report we show a specific gene expression profile for WSE that involves 57 genes. Using biological or functional network analysis, 37 genes were identified around UBC, GABARAPL1 genes and PI3K/AKT and MEK/ERK signaling pathways.

The UBC hub in Network 1 (Fig. 4) is involved in cellular homeostasis and signaling. It was originally activated to degrade misfolded or disused proteins, but it has been recently associated with the cell cycle, DNA repair, endocytosis, antigen processing and apoptosis [34]. Recently, Tang et al. demonstrated that the inhibition of the ubiquitin system decreased the proliferation and radio-resistance in the H1299 cell line (NSCLC cells) [35]. In this regard, a clinically relevant observation is the approval of bortezomib as an inhibitor of the protein degradation system in human cancer [32].

The GABARAPL1 hub in Network 2 (Fig. 5) is a highly conserved protein throughout evolution. It is related to autophagy and vesicle intracellular transport [36]. Its participation in cancer is still not clear, but it has been reported that lower levels of this transcript correlates with decreased survival in patients with neuroblastoma [37] and increased metastasis in breast cancer [36]. On the other hand, the ectopic over-expression of GABARAPL1 inhibits cancer cell proliferation and tumor growth in mice [38]. There are other reports that relate low expression of this gene in several cancer cell lines [39].

Regarding the last network, there have been reports that show that PI3K/AKT and MEK/ERK signaling pathways are altered in NSCLC and their activation is associated with malignant transformation and drug resistance (Figs. 6 and 7). MEK and PI3K inhibitors can inhibit cell proliferation in NSCLC; however, for apoptosis activation, both signaling pathways must be simultaneously inhibited [40, 41], a situation that is directly related to the frequently observed *EGFR*-TKI resistance in this tumor. There are other reports showing that *EGFR* mutations function as inductors to sensitization to TKIs through PI3K/AKT and MEK/ERK signaling pathways [41–45]. It has also been demonstrated that cases with *EGFR* mutations have a major sensibility to the *EGFR*-TKIs, using inhibitors from PI3K/AKT and MEK/ERK [41–45]. On a clinical note, our group has previously reported the association between NSCLC adenocarcinoma and positive *EGFR* mutation status in patients with history of WSE compared to tobacco smoke exposure.

WSE is also related to gene promoter methylation that synergistically increases the risk for reduced lung function in cigarette smokers [46]. A recent report describing the toxicological characteristics associated with WSE in A549 cell lines, including high levels of polycyclic aromatic hydrocarbons (PAH) and low level of water-soluble metals, showed an enhanced level of free radicals, DNA damage and the major expression of inflammatory/oxidative stress genes [47]. There is evidence that some potential molecular targets, such as *EGFR* and the ErbB family receptor, are usually altered in epithelial tumors [48]. *EGFR* mediates cell proliferation, differentiation, survival, angiogenesis and migration, and is overexpressed in approximately 40–80 % on NSCLC tumors [49–51].

Clinically, it is known that EGFR inhibitors in NSCLC extend survival after first-line or second-line therapy in patients with EGFR mutations [52]. These mutations are more frequent in specific populations, including women, Asian and Hispanic ethnicities, never-smokers and adenocarcinoma histology [18, 53, 54]. Activating mutations in EGFR leads to constitutive tyrosine kinase activation and oncogenic transformation of lung epithelial cells [12, 13]. In this sense, the presence of these common activating EGFR mutations is tightly associated with sensitivity to reversible EGFR- specific tyrosine kinase inhibitors (e.g.: erlotinib or gefitinib). Patients with these mutations display EGFR-TKIs response rates of approximately 70 % a median progression free survival (PFS) of approximately 9–12 months and overall survival rates that may exceed 20–32 months [55]. Most patients will experience disease progression and drug resistance attributed to the development of other second mutations or with the presence of other uncommon EGFR mutations [56]. Certain therapeutic relations in NSCLC include the main oncogenic protein KRAS-GTP with biological significance between EGFR and PI3K/AKT or MEK/ERK pathways [56]. The presence of KRAS mutations leads to an increased signal through the MEK/MAPK transduction pathway [56]. Rare cases of mutations of MEK have been reported in NSCLC [57]. Preclinical studies in both the KPC mouse model as well as patient-derived xenografts have shown that blocking the MAPK pathway at MEK results in a decrease of cell proliferation and a subsequent halt in tumor growth [58]. The activation of EGFR recruits PI3K to the cell membrane and phosphorylates phosphatidylinositol-2-phosphate (PIP2) to phosphatidylinositol-3-phosphate (PIP3), which in turn activates AKT and several downstream effectors [59]. Inhibitors of both PI3K and AKT have been developed [60], although inhibition of PI3K is complicated by the fact that there are multiple isoforms of the protein [61]. Another biological interaction takes place on KRAS is one that directly activates PI3KCA [62]. Unlike most oncogenic driver mutations on NSCLC, PI3K mutations may occur in association with EGFR or KRAS mutations [63]. Although rare, PI3K/AKT/mTOR pathway activation may occur through AKT mutations in NSCLC [64]. Clinical evidence has shown that reversible EGFR-TKIs are considered the frontline treatment for advanced NSCLC patients harboring EGFR mutations [65]. New emerging evidence suggests that the anti-tumor activity of EGFR-TKIs in resistant NSCLC cell lines can be enhanced by combined therapy with other regimens. Early efforts have shown that cetuximab, produced synergistic anti-proliferative effects when used in combination with gefitinib or erlotinib [66]. Our analyses provide biological networks relationships between 37 genes and PI3K/Akt and MEK signaling for understanding the biologic properties of WSE effects as a carcinogenic factor in NSCLC. It also shows useful common pathway maps for a future understanding of the disease and the development of new therapeutic targets.

Whilst the differences in gene expression patterns between WSE or tobacco-related lung cancer that we identified in this paper provide an important insight into the molecular basis of the clinical and biological differences between these two tumors, there is a limitation regarding the small sample size. However, this is countervailed by a thorough characterization of the samples, a detailed clinical history and close follow-up on all patients. It is imperative to continue further study to validate the potential biological and clinical implications of our findings.

## Conclusion

In conclusion, our results suggest a differential gene expression profile for WSE or tobacco-related lung cancer, which suggests different carcinogenesis mechanisms between both risk factors and enlightens the clinical-pathological and mutational profiles between both groups with adenocarcinoma.
